# Initial loss to follow up among tuberculosis patients: the role of Ward-Based Outreach Teams and short message service (SMS) technology (research proposal)

**DOI:** 10.1186/s13104-019-4757-3

**Published:** 2019-11-08

**Authors:** Judith R. M. Mwansa-Kambafwile, Charles Chasela, Nazir Ismail, Colin Menezes

**Affiliations:** 1 Department of Public Health, University of Witwatersrand, Johannesburg, South Africa; 2 Centre for Tuberculosis, National Institute of Communicable Diseases, Johannesburg, South Africa; 3 Consortium for Advanced Research Training in Africa (CARTA), Nairobi, Kenya; 4 Department of Microbiology, University of Pretoria, Pretoria, South Africa; 5 Department of Internal Medicine, School of Clinical Medicine, Faculty of Health Sciences, University of Witwatersrand, Johannesburg, South Africa; 6 Department of Internal Medicine, Chris Hani Baragwanath Academic Hospital, Johannesburg, South Africa

**Keywords:** TB, Initial LTFU, WBOTs, SMS, Messaging

## Abstract

**Introduction:**

Tuberculosis (TB) is a problem in South Africa. Initial loss to follow up (LTFU) among TB patients is high varying between 14.9 and 18%. Some of the reasons for this are: lack of proper communication between patient and staff on next steps after testing, not aware that results are ready; and other competing priorities. Receiving reminder messages that result is ready is an intervention that can be explored to reduce initial LTFU. This can be through either receiving a note from the Ward-Based Outreach Teams (WBOTs) or via short message service (SMS) advising the patient to collect test result at the facility. This proposal aims to assess the effectiveness of WBOTs or SMS technology in reducing TB initial LTFU.

**Methods:**

This will be a mixed methods approach. In depth interviews with WBOT Managers and TB Program Managers will be conducted. Focus group discussions with WBOT members will also be conducted. Two interventions (enhanced WBOTs/SMS technology) will be tested using a 3 arm randomized controlled trial (standard of care, SMS technology or enhanced WBOTs). The WBOTs will deliver paper note reminders while SMS intervention will entail sending reminder SMS messages to patients as soon as TB results are ready.

## Introduction

Tuberculosis (TB) is a deadly communicable disease, killing approximately 1.6 million of the global 10 million people who developed the disease in 2017. South Africa’s TB incidence is currently 567/100,000 population whilst the HIV prevalence among these incident cases is 60% [1]. Although the mortality due to TB in South Africa has been declining, the disease still tops the list of the “ten leading underlying natural causes of death, 2014–2016” [2].

Patients whose TB test results are positive but never get initiated on treatment are known as initial loss to follow up patients.

There are different reasons why patients diagnosed with TB do not start treatment. Some of the reasons documented are those written in registers such as “transfer out” or “died”. In a Malawian study, critical narrative interviews with 19 of 23 patients not initiated on treatment revealed that lack of education and poverty were the main characteristics of these patients [3]. A later study conducted in Pakistan corroborates these findings and in addition found that not having someone to go with to the clinic, consulting traditional healers, social stigma and religious beliefs contributed to initial LTFU [4].

Apart from the patient centred factors highlighted above, Botha et al. found that more frequent causes for initial LTFU are those to do with the quality of the healthcare services rendered to these patients. They found that at least 56% of the TB patients they interviewed after tracing them gave reasons for LTFU directly linked to the services at the facilities [5]. The long pathways to diagnosis, characterized mainly by health system structural barriers were also a cause for initial LTFU in the Malawian study [3]. Lack of proper recording and reporting in facilities can result in an overestimate of initial LTFU as was found in a study conducted in India [6].

A systematic review which assessed the magnitude of the initial LTFU rate in smear-positive or culture-positive TB patients found that this rate varies between 4 and 38% with weighted values for African studies of 18% and for Asia of 13% [7]. A more recent study conducted in South Africa reported pre-treatment LTFU rates of 14.9 and 17.0% for Xpert and smear microscopy respectively; this was despite telephonic or home visit contact by study investigators at week 1 and 1 month into the trial [8]. Another South African study conducted in Kwa Zulu Natal province found an initial LTFU rate of 17.9% from retrospective review of TB case identification registers for the year 2007 [9].

TB patients already on treatment have been a focus of TB control programs in many countries. The emphasis has mostly been on ensuring that patients take their treatment. This has been evidenced from the implementation of Directly Observed Therapy Short-Course (DOTS). In addition, the scope of work for the Ward-Based Outreach Teams (WBOTs) of the re-engineered primary healthcare model includes adherence support to TB patients on treatment. However, not much attention has been paid to the patients who test positive for TB but never get initiated on treatment. TB patients who are lost before treatment is initiated continue to transmit infection in communities. This is an important group of people as they contribute to the burden of TB in communities. Treatment initiation is a way of infection control because a patient on treatment stops being infectious within 2 weeks of starting treatment [10, 11].

Sending reminders to patients to inform them that their results are ready at the facility where they tested could help increase the number of TB patients initiating treatment. Two ways these reminders could be sent are through short message service (SMS) messaging to patients or through paper notes delivered to patients by the WBOTs. This study intends to assess the effectiveness of WBOTs and SMS technology to reduce initial LTFU among TB patients. More detail about the WBOT and SMS is provided below.

### Ward-Based Outreach Teams (WBOTs)

Within the structure of South Africa’s re-engineered PHC model are WBOTs. These work within specific geographical areas of PHC facility total catchment areas. The WBOTs consist of a team leader (often a Professional Nurse) and 6 community healthcare workers (CHWs) including a health promoter (HP) and an environmental health officer (EHP) where these exist [12]. The WBOT is linked to a PHC facility through the Outreach Team Leader who is usually a Professional Nurse based at a PHC facility. The objective of this stream of the PHC model is to provide PHC services at household and community levels. In Brazil, the model of taking healthcare services to the communities resulted in improved access to healthcare among the people and consequently healthier communities [13]. The findings of an evaluation of Brazil’s Family Health Program on infant mortality rate show a decline from 49.7/1000 live births in 1990 to 28.9/1000 live births in 2002 [14]. The WBOTs refer clients to appropriate PHC facilities when necessary thus linking them to healthcare. The scope of work is centred on health promotion and prevention of disease (Table 1 [15]). With regards to TB services, the WBOTs’ work is to identify, support and follow-up TB patients and their contacts [12]. They are supposed to ensure that TB patients adhere to treatment once initiated [16]. An exploratory research assessing TB/HIV-related training, knowledge and attitudes of CHWs conducted in both urban and rural parts of the Free State Province of South Africa revealed that at least a third of the CHWs had not been trained on basic TB/DOTs and over half of them had no formal training on HIV counselling and testing [17]. An earlier study conducted in Papua New Guinea on CHW clinical competency showed that CHW obtained low scores on clinical knowledge/skills and these scores worsened with duration of time since last training. The authors recommended closer on-site supervision as well as semi-annual trainings to ensure optimal healthcare service delivery by this cadre of healthcare workers [18].

**Table 1:** Scope of work for CHW on the WBOTs. Source: Provincial guidelines for the implementation of the three streams of PHC re-engineering [15]

Improve the quality of life of community members by mobilizing for improved access to and delivery of Primary Health Care at local level within the context of an inter-sectoral environment	
1	Promote health and prevent illness
2	Conduct community assessments and mobilize around community needs
3	Conduct structured household assessment to identify their health needs
4	Provide psychosocial support to community members
5	Identify and manage minor health problems
6	Support screening and health promotion programmes in schools and Early Childhood Development (ECD) centers
7	Promote and work with other sectors and undertake collaborative community based interventions
8	Support continuum of care through service coordination with other relevant service providers

### Short message service (SMS) Technology

Globally, mobile cellular phone usage has grown tremendously over the past decade. By 2015, there were over 7 billion mobile cellular subscriptions [19]. Use of short message service (SMS) technology in patient healthcare has resulted in favourable outcomes particularly with regards to adherence to various chronic medications [20–23]. This technology is also acceptable among patients who use it [16, 24]. A systematic review which looked at interventions used to promote adherence to ART among HIV infected individuals found that there was a 65% increased chance of adherence to ART (confidence interval = 1.25–2.18) when SMS messages were used compared to no SMS [25].

SMS messaging has also been used to improve adherence to treatment among patients TB patients. Different companies have developed techniques to improve adherence to TB treatment. One of these techniques (SIMpill technique) involves the packaging of TB drugs in a special bottle that has a sim card in it. Once this bottle is opened, a message gets sent to a central server which stores the unique information for that medication bottle. This is linked to the patient’s name and contact details. If the bottle is not opened, an SMS reminder gets sent to the patient’s phone number. If the bottle is still not opened, another SMS is then sent to the patient’s alternative number (relative’s phone number). This SIMpill was piloted among 155 TB patients between July 2006 and April, 2007. The findings show improvement in both adherence and treatment success rate of 86–92% and 94% respectively [26]. Sending SMS messages to TB suspects who test positive asking them to go to the clinic could potentially contribute to reduction in initial LTFU among TB patients.

A study conducted in Cambodia which looked at active TB case finding in communities used SMS technology to send positive test results from the laboratory to TB workers. The TB workers would then inform the respective patients either telephonically or through Community Health Volunteers who conducted home visits. The researchers found that 94.6% (741/783) of the patients diagnosed with TB were initiated on treatment and at a median time of 3 days (IQR 1–6) [27].

Although linkage to care has been a favorable outcome of using SMS intervention in healthcare, there have also been instances of unresponsiveness to SMS messages. A study to understand the factors which influence adolescents’ non-responsiveness to text messaging was conducted by Irons et al. [28]. This was a sub-study of a trial that evaluated the feasibility, acceptability and effectiveness of a text messaging reminder system to improve clinic attendance at family planning appointments among 5 young women using Depo-Provera contraception. The researchers found that personal conflicts such as school or work were a main cause for non-responsiveness. Findings from this study, however, cannot be generalized. The sample was drawn from the intervention arm of a small feasibility and acceptability family planning trial. The sample size may have limited ability to further stratify to identify differences between non-responders [28].

### Problem statement

According to the South African National TB guidelines, treatment success rate is the proportion of new smear positive TB patients cured plus the number completed treatment but not meeting the criteria for “cure” or “failure”. The denominator for this is the total number of new smear positive pulmonary TB patients registered [29]. However, the number of patients starting treatment is only a proportion of the ones eligible [7, 30]. There is a possibility that the success rate of the TB control program is an overestimate due to the assumption that the initial LTFU rate is negligible and so not factored in. Initial LTFU rates in South Africa range between 14.9 and 18% [8, 9]. These rates are much higher than the national target of “less than 5%” for the South African TB Control Program [29]. Long pathways to diagnosis, characterized mainly by health system structural barriers, have been found as one of the reasons for initial LTFU [3]. There is need to have ways that will help reduce this rate so as to achieve the national target.

### Study justification

Adherence support to TB treatment in patients already initiated on treatment is covered in the scope of work of the WBOTs. However, following up patients diagnosed with TB but not initiated on treatment is not. TB treatment in a patient rapidly decreases infectivity, decreases transmission and is vital for TB control [10, 11]. Delaying TB treatment initiation or losing bacteriologically confirmed TB patients before treatment is initiated contributes to on-going TB transmission in communities and to poor patient outcomes. Strategies to reduce initial LTFU are needed in order to reduce on-going transmissions in communities. WBOTs are an important cadre of workers who make it possible for healthcare services to be delivered at the family and community levels. The concept of community level healthcare has been shown to work in reducing infant mortality rate [14]. Treatment adherence to chronic medication such as TB and HIV treatment is also done by the WBOTs [16]. However, ensuring that TB patients are initiated on treatment is not part of their scope of work. Having WBOTs involved in ensuring treatment initiation among TB patients is one way of reducing initial LTFU. SMS technology has also been shown to work in different programs [26]. Sending SMS messages to patients testing positive for TB is another way.

### Conceptual framework

**Fig. 1:**
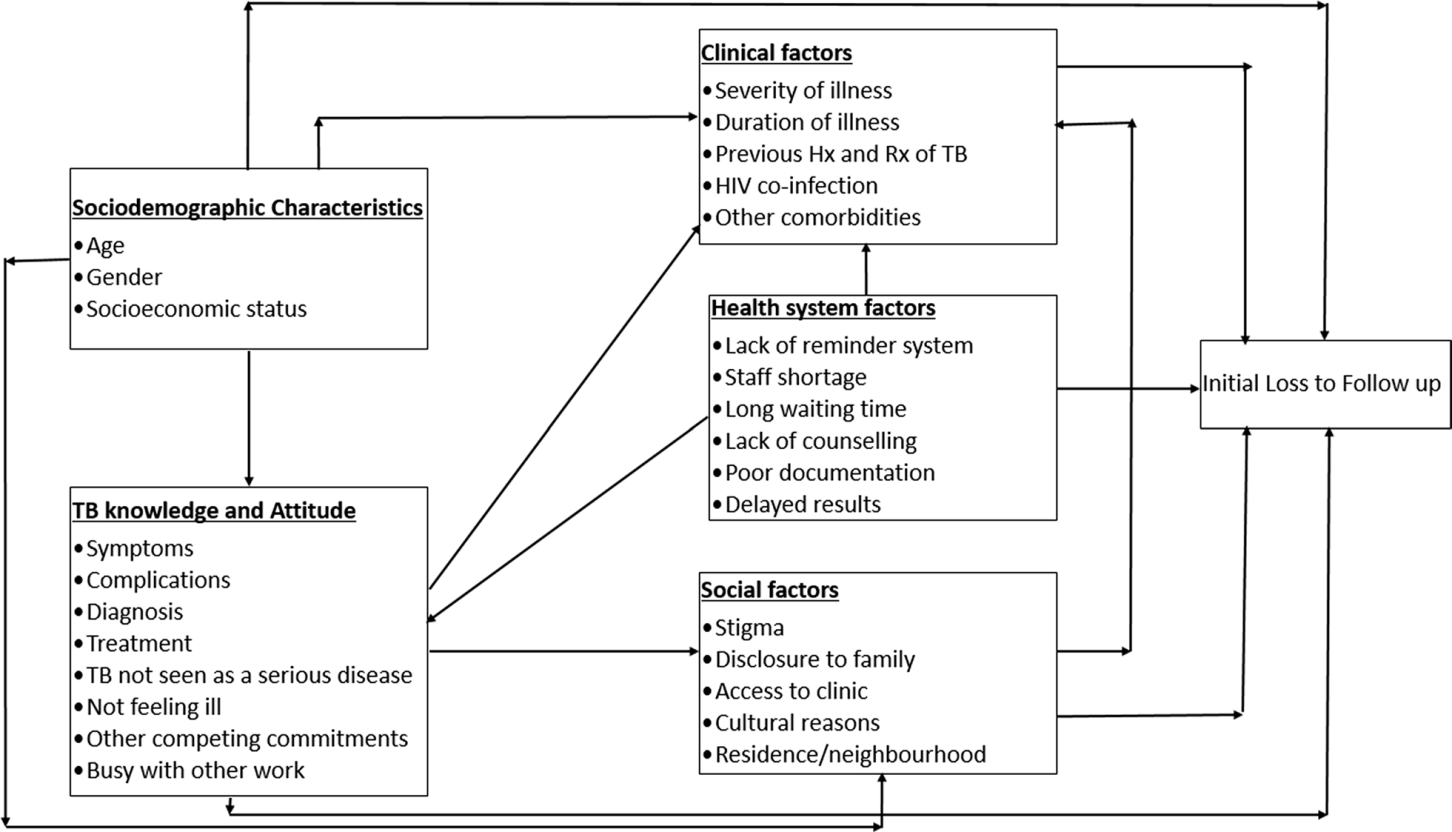
Conceptual framework for TB treatment initiation

The conceptual framework for this study (Fig. 1) is based on a combination of 2 conceptual models. The Health Belief Model of the 1950s and later modified by Rosenstock et al. gives possible concepts to explain health behaviour [31]. The model by Krishnan et al. looks at individual and provider/system barriers/delays to TB diagnosis and treatment at various time points along the continuum of TB care [32]. The framework above takes into account the fact that there are both patient and health system factors that can prevent a patient from starting treatment. SMS and paper note reminders will address both patient and health system factors. Both methods will remind a patient that he/she needs to find time to go and collect the test results at the clinic. With regards to health system factors, WBOTs are a cadre of staff that can be utilized to ensure that patients return to the clinic for their results. This will reduce the proportion of patients diagnosed with TB but not initiated on treatment thereby reducing the TB transmission in communities.

#### Aim

The main aim is to assess the effectiveness of WBOTs and/or SMS technology in reducing initial LTFU among TB patients.

#### Objectives

1. To determine reasons for initial LTFU from the perspective of TB Program Managers and WBOT Managers.
2. To understand the nature of the work of WBOTs (spread, their reach, potential limitations they foresee with TB follow up work).
3. To assess the effectiveness of WBOTs in reducing initial LTFU among TB patients.
4. To assess the effectiveness of SMS technology in reducing initial LTFU among TB patients.
5. To describe the implementation process of distribution of paper note and SMS reminders to patients testing for TB from the perspectives of TB patients and of WBOTs.

## Methods

**Fig. 2:**
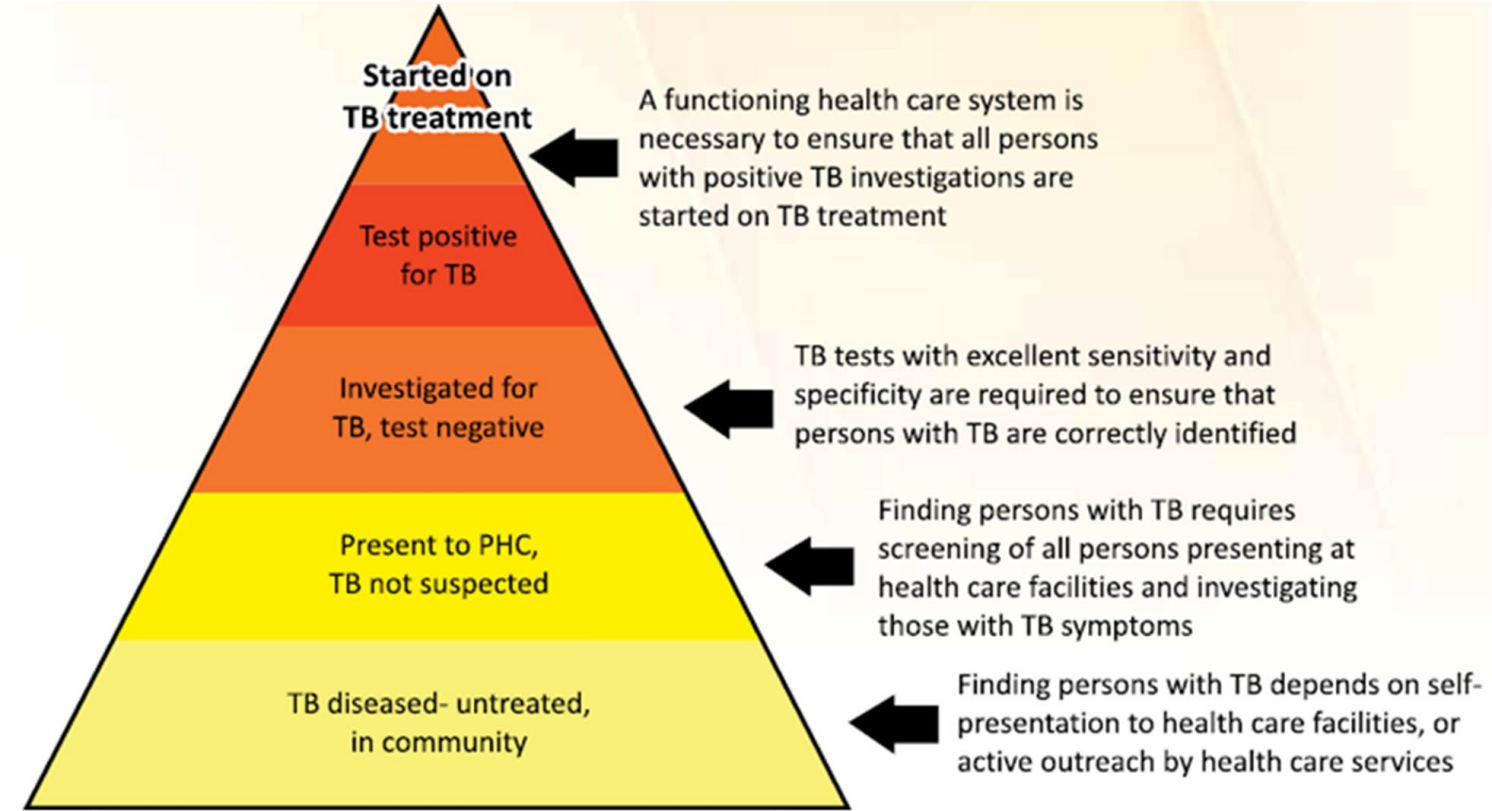
Steps required for the diagnosis of TB (Source: SA National Tuberculosis Guidelines 2014 [29])

The current cascade for patients with presumptive TB (TB suspects) in South Africa is as shown in Fig. 2 [29]. A patient feeling sick and presenting to a primary healthcare (PHC) facility is screened for TB symptoms. If productive cough is among the presenting symptoms, the patient is asked to produce sputum on the spot. This sputum is sent to the laboratory for testing. In the laboratory, the sputum is tested using the Xpert MTB/Rif (Xpert) machine if available. If not available, smear microscopy is the diagnostic test used. Laboratory turnaround time for a test using the Xpert machine is 2 h. The patient is asked to come back after 2 days to cater for transportation time and delivery of result to the PHC facility. When the patient returns and if TB test result is positive, treatment is initiated same day or within 5 days. If diagnosis was done using Xpert, the patient is asked to submit another sputum sample for smear microscopy. This second sample is needed as a baseline for monitoring treatment progress when changing from intensive phase to continuation phase of treatment and at time of treatment completion. Xpert is not used for monitoring as it tends to pick up dead TB bacilli and would therefore give a positive test result even after 2 months of treatment [29].

The methods are described in detail below based on respective objectives.

### Objective 1: To determine reasons for initial LTFU from the perspective of TB Program Managers and WBOT Managers

Study design: In depth interviews with TB Program Managers and WBOT Managers.

Study setting: City of Johannesburg.

Study population: TB Program Managers and WBOT Managers.

Sample size and data collection: In-interviews using a structured interview guide will be employed. Purposive sampling for maximum variation will be employed until saturation is reached. The interviewers will be researchers with experience in conducting in-depth interviews and with no relationship with potential participants. The interviews will be audio-recorded and then transcribed in readiness for analysis. To ensure reliability of the coding framework, the transcripts will be reviewed by 2 people so as to agree on the framework.

Outcomes: An understanding of the problem of initial LTFU of TB patients from the perception of managers. Thematic analysis in Nvivo 11 software will be used to analyse the data.

### Objective 2: To understand the nature of the work of WBOTs (spread, their reach, potential limitations they foresee with TB follow up work)

Study Design: In depth interviews/Focus group discussions with WBOT members.

Study setting: City of Johannesburg.

Study population: Members of the WBOTs.

Sample size and data collection: Members of the WBOTs in the study area will be approached for participation. Purposive sampling for maximum variation will be employed until saturation is reached. A structured interview guide will be used.

Outcomes: An understanding of the nature of the work of WBOTs. Thematic analysis in Nvivo 11 software will be used to analyse the data.

### Objectives 3 and 4: To assess the effectiveness of SMS messaging or WBOTs on initial LTFU among TB patients

These 2 objectives are covered under the same methods.

Study design: This will be an Individual Randomised Controlled Trial. Two interventions (enhanced WBOTs and SMS technology) will be tested using the same population. Therefore, there will be 3 arms at the different sites/facilities (each site implementing all 3 arms of the study). The intervention “Enhanced WBOTs” refers to WBOTs conducting delivering paper note reminders to patients whose test results are ready in addition to their current scope of work [12]. SMS intervention will entail sending reminder SMS messages to TB patients as soon as results are received at the facility.

Arm A: Standard of care (no SMS technology and no enhanced WBOTs).

Arm B: SMS technology.

Arm C: Enhanced WBOTs.

A summary of the protocol is shown in Table 2.

**Table 2:** Research protocol summary

Objective	Study design	Study population, sample and sample size	Data collection tools/method	Main outcome variables/construct	Data analysis technique
To determine reasons for initial LTFU from the perspective of service providers	In-depth interviews	TB Program Managers/WBOT Managers (min 6)	Structured interview guide	Knowledge of the TB program; reasons for initial LTFU	Thematic analysis
To understand the nature of the work of WBOTs (spread, their reach, potential limitations they foresee with TB follow up work)	In-depth interviews/Focus group discussion	WBOT members from facilities with functional WBOTs (min 4)	Structured interview guide	Knowledge of the work of the WBOTs	Thematic analysis
To determine the effectiveness of WBOTs in reducing initial LTFU among TB patients	Randomized controlled trial	Patients testing for TB TB positive patients (312)	Data from TB case identification register and from TB treatment initiation registers to a study data abstraction form	Treatment initiation within 4 weeks of submitting sputum for diagnosis	Descriptive analysis Chi square statistics regression analysis (cox and multinomial)
To determine the effectiveness of SMS technology in reducing initial LTFU among TB patients	Randomized controlled trial	Patients testing for TB TB positive patients (312)	Data from TB case identification register and from TB treatment initiation registers to a study data abstraction form	Treatment initiation within 4 weeks of submitting sputum for diagnosis	Descriptive analysis Chi square statistics regression analysis (cox and multinomial)
To describe the implementation process of distribution of paper note and SMS reminders to patients testing for TB from the perspectives of TB patients and of WBOTs	Descriptive case study	TB patients (min 6) WBOTs (min 6)	In-depth interviews	Perceptions of TB patients and those of WBOTs of the process of distribution of reminder messages	Thematic analysis

Study setting: Inner-city Johannesburg metropolitan area in South Africa occupies an area of 1645 km^2^ and has a population of 4.4 million [33]. WBOTs focus on health promotion, disease prevention and adherence support [12, 16]. Enhanced WBOTs will not only focus on those 3 areas, but they will also ensure that patients diagnosed with TB are initiated on treatment.

Study population: Patients with presumptive TB accessing healthcare services from the PHC facilities.

Inclusion criteria for TB suspects: Patients aged 18 years old and above not yet diagnosed with TB who present with productive cough of more than 24 h.

Exclusion criteria for TB suspects: Children less than 18 years old; patients already on TB treatment.

### Sampling and sample size calculation

Eight high burden facilities with functional WBOTs linked to them will be conveniently selected from the health facilities in the district.

Patients meeting the inclusion criteria at the selected facilities will be allocated to any of the 3 arms. This will be by permuted block randomization. Patients who test positive for TB will be allocated any of the three letters from A to C depending on a pre-run block randomization sequence. The letter on the paper indicates the arm to which the patient is allocated to.

Due to lack of literature on SMS messaging and use of WBOTs to reduce initial LTFU in TB services, it will be assumed that the initial LTFU in this study will decrease from the 18% upper limit reported in South African studies to 5% in each of the arms with an intervention (with either SMS technology or with enhanced WBOTs). Sample size calculation will be calculated for either intervention versus control (standard of care). With a power of 80% and a level of significance of 0.05, the sample size required will be 94 TB patients in each arm. This gives a total sample of 282 for the 3 arms together. Accounting for 10% LTFU, the total minimum sample size of 311 TB patients will be required. Therefore, a sample size of 312 with 104 in each arm will be taken.

### Interventions

The interventions are described below in detail for the different arms of the study.

#### Arm A: Standard of care (No enhanced WBOTs and no SMS technology)

As per current standard of practice, patients with presumptive TB submitting sputum will be asked for their contact details including mobile phone numbers. This information will be entered in the TB Case Identification Register as well as in the study book. The patients will submit sputum and asked to collect test result after 2 days. The TB Case Identification Register at the clinic will be checked regularly to check the results of the patients enrolled in the study. The names of those with a positive result will be checked for in the TB Treatment initiation register. The TB patients will be allocated to this arm if they are allocated the letter “A” from the randomization block. After 4 weeks, the ones with positive TB test result but not initiated on treatment will be noted.

#### Arm B: SMS technology

This group will have the SMS technology as the intervention. As per current standard of practice, patients with presumptive TB submitting sputum will be asked for their contact details including mobile phone numbers. This information will be entered in the TB Case Identification Register as well as in the study book. The patients will submit sputum and asked to collect test result after 2 days. Patients with positive test results will receive SMS messages telling them that their results are ready at the facility. The TB patients’ names will be allocated to this arm if they are allocated the letter “B” from the randomization block. The TB treatment initiation register at the clinic will be checked regularly for names of the patients randomized to this arm for a period of 4 weeks. The ones with positive TB test but not initiated on treatment will be noted.

#### Arm C: Enhanced WBOTs

As per current standard of practice, patients with presumptive TB submitting sputum will be asked for their contact details including mobile phone numbers. This information will be entered in the TB Case Identification Register as well as in the study book. The patients will be informed that results of the test will be ready after about 2 days when they can go to the clinic to collect them. The patients’ names who test positive for TB and allocated to this arm will be given to the WBOTs. The patients will be allocated to this arm if they are letter “C” from the randomization block. The WBOTs will then deliver a paper note advising the patient to go to the clinic to collect TB results. In the event that the WBOTs do not find the patient at the named address, they will leave the paper note. Treatment initiation registers at the clinics will be checked regularly for a period of 4 weeks. The ones with positive TB test result but not initiated on treatment will be noted.

Outcome: The primary outcome is the proportion of TB patients not initiated on treatment within 4 weeks of submitting sputum for diagnosis among patients accessing services from the 3 study arms. A secondary outcome will be the time to treatment initiation.

Analysis: Descriptive analysis to determine the initial LTFU for the different study arms will be run and comparisons across the different arms will be done using Chi square statistics and regression analysis (cox and multinomial) to determine associations and predictors of initial LTFU.

### Objective 5: To describe the implementation process of distribution of paper note and SMS reminders to patients testing for TB from the perspectives of TB patients and of WBOTs

Study design: Focus group discussions and/or in-depth interviews with WBOT members and In-depth interviews with some of the TB patients who received reminder messages.

Study setting: City of Johannesburg.

Study population: Members of the WBOTs at the study sites and also TB patients who received reminder messages.

Inclusion criteria: WBOT members at the study sites and TB patients who were part of the study.

Exclusion criteria: WBOT members from facilities not from the study sites and TB patients not enrolled in the study.

### Sample size and data collection

All available members of the WBOTs at the study sites will be approached for participation in a focus group discussion or in-depth interviews will be conducted if the WBOT members are not available at the same time for a focus group discussion. Focus group discussion/interview guide will be used. In-depth interviews with TB patients using a structured interview guide will be employed. Purposive sampling for maximum variation will be employed until saturation is reached. The interviewers will be researchers with experience in conducting in-depth interviews and with no relationship with potential participants. The interviews will be audio-recorded and then transcribed in readiness for analysis. To ensure reliability of the coding framework, the transcripts will be reviewed by 2 people so as to agree on the framework.Outcomes: Perceptions of the implementation process by implementers (WBOTs) and the by the users (TB patients). Thematic analysis in Nvivo 11 software will be used to analyse the data.

## Limitations

A main limitation with sending SMS messages will be that there will be no way of ascertaining if the messages reach the targeted people.

Some patients might not go to collect their test result at the facility where the TB test was done. They might test elsewhere and actually get initiated on TB treatment. To overcome this, contact details of participants will be collected so that after the 1 month period, it will be possible to know if the patients are indeed LTFU by checking treatment registers in the facilities within inner-city Johannesburg as well as the ETR.net/TIER.net.

## Data Availability

This article is a research proposal. The data collected thus far has not yet been analysed.

## Abbreviations

TB: tuberculosis
LTFU: loss to follow up
WBOT: Ward-Based Outreach Team
SMS: short message service
WHO: World Health Organization
DOTS: Directly Observed Therapy Short-Course
ART: antiretroviral therapy
Xpert: Xpert MTB/Rif
PHC: primary healthcare
CHWs: community healthcare workers
HP: health promoter
EHP: environmental health officer
